# The Sfp-Type 4′-Phosphopantetheinyl Transferase Ppt1 of *Fusarium fujikuroi* Controls Development, Secondary Metabolism and Pathogenicity

**DOI:** 10.1371/journal.pone.0037519

**Published:** 2012-05-25

**Authors:** Philipp Wiemann, Sabine Albermann, Eva-Maria Niehaus, Lena Studt, Katharina W. von Bargen, Nelson L. Brock, Hans-Ulrich Humpf, Jeroen S. Dickschat, Bettina Tudzynski

**Affiliations:** 1 Institut für Biologie und Biotechnologie der Pflanzen, Westfälische Wilhelms-Universität Münster, Hindenburgplatz 55, Münster, Germany; 2 Institut für Lebensmittelchemie, Westfälische Wilhelms-Universität Münster, Corrensstraße 45, Münster, Germany; 3 Institut für Organische Chemie, Technische Universität Braunschweig, Hagenring 30, Braunschweig, Germany; Seoul National University, Republic of Korea

## Abstract

The heterothallic ascomycete *Fusarium fujikuroi* is a notorious rice pathogen causing super-elongation of plants due to the production of terpene-derived gibberellic acids (GAs) that function as natural plant hormones. Additionally, *F. fujikuroi* is able to produce a variety of polyketide- and non-ribosomal peptide-derived metabolites such as bikaverins, fusarubins and fusarins as well as metabolites from yet unidentified biosynthetic pathways, e.g. moniliformin. The key enzymes needed for their production belong to the family of polyketide synthases (PKSs) and non-ribosomal peptide synthases (NRPSs) that are generally known to be post-translationally modified by a Sfp-type 4′phosphopantetheinyl transferase (PPTase). In this study we provide evidence that the *F. fujikuroi* Sfp-type PPTase FfPpt1 is essentially involved in lysine biosynthesis and production of bikaverins, fusarubins and fusarins, but not moniliformin as shown by analytical methods. Concomitantly, targeted Ff*ppt1* deletion mutants reveal an enhancement of terpene-derived metabolites like GAs and volatile substances such as α-acorenol. Pathogenicity assays on rice roots using fluorescent labeled wild-type and Ff*ppt1* mutant strains indicate that lysine biosynthesis and iron acquisition but not PKS and NRPS metabolism is essential for establishment of primary infections of *F. fujikuroi*. Additionally, FfPpt1 is involved in conidiation and sexual mating recognition possibly by activating PKS- and/or NRPS-derived metabolites that could act as diffusible signals. Furthermore, the effect on iron acquisition of Ff*ppt1* mutants led us to identify a previously uncharacterized putative third reductive iron uptake system (FfFtr3/FfFet3) that is closely related to the FtrA/FetC system of *A. fumigatus*. Functional characterization provides evidence that both proteins are involved in iron acquisition and are liable to transcriptional repression of the homolog of the *Aspergillus* GATA-type transcription factor SreA under iron-replete conditions. Targeted deletion of the first *Fusarium* homolog of this GATA-type transcription factor-encoding gene, Ff*sre1*, strongly indicates its involvement in regulation of iron homeostasis and oxidative stress resistance.

## Introduction

**Figure 1:**
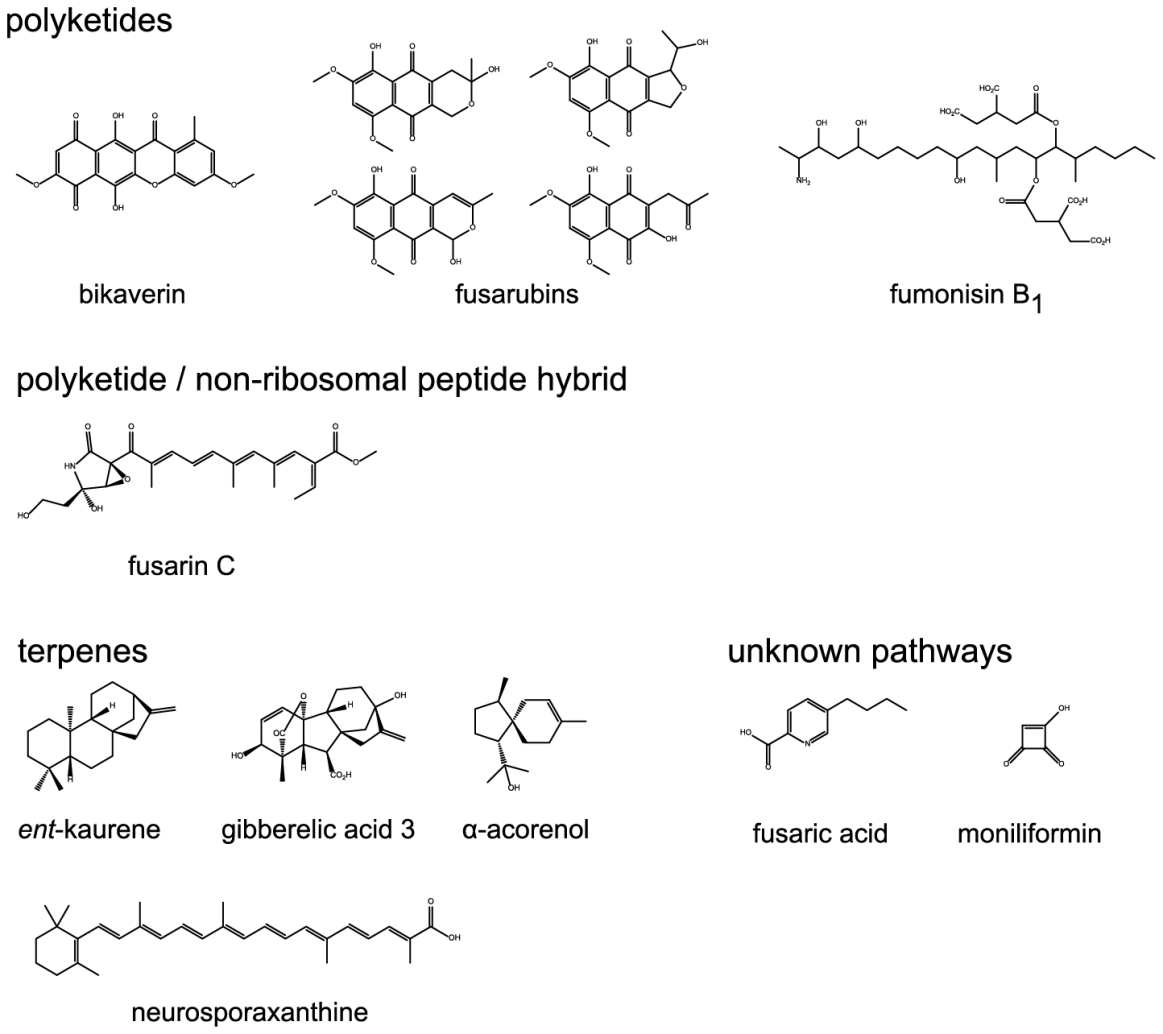
Known secondary metabolites of *F. fujikuroi*. Known secondary metabolites produced by *F. fujikuroi* classified by their biosynthetic pathways.

Filamentous fungi of the genus *Fusarium* are notorious pathogens of economically relevant crops. They produce a variety of bioactive secondary metabolites (Fig. 1) that pose a potential threat to animals and humans when consumed. In particular, the well known rice pathogen *F. fujikuroi* is able to produce *ent*-kaurene-derived gibberellins (GAs) [1], bikaverin [2], neurosporaxanthin [3], fusarin C [4], fusaric acid [5], moniliformin [6], fumonisins [7], α-acorenol [8], and fusarubins [9] (Fig. 1). Some of these substances have harmful effects on human cell lines, e.g. bikaverin, fusarins, and fumonisins [10]–[13] and in animal models, e.g. moniliformin [14]. Other metabolites play a role as virulence factors in fungal-plant interaction, e.g. fusaric acid, fumonisins, and GAs [15]–[17]. The latter belong to a class of isoprenoid phytohormones that are secreted by the fungus thereby causing the *bakanae* or “foolish seedling” disease of rice. The afflicted plants are visibly etiolated and chlorotic, do not produce edible grains, and are incapable of supporting their stem weight at late stages of the disease [18]. Beside this disease-causing action, some GAs are used in agriculture, viticulture, and horticulture as important plant growth regulators which are largely produced by submerged fermentation of the fungus *F. fujikuroi* on an industrial scale [19].

The secondary metabolites produced by filamentous fungi can be classified into distinct chemical groups as polyketides, non-ribosomal peptides, chimeric molecules composed of a polyketide and a non-ribosomal peptide moiety, terpenes, and (prenylated) alkaloids. Production of secondary metabolites of each group involves specific key enzymes, hence named polyketide synthases (PKSs), non-ribosomal peptide synthetases (NRPSs), PKS/NRPS hybrids, terpene cyclases (TCs), and prenyl transferases (PTs) [20]. The recently sequenced genome of *F. fujikuroi* strain IMI58289 identified the existence of genes encoding 13 type I PKSs, 1 type III PKS, 11 NRPSs, 3 PKS/NRPS hybrids, 8 TCs, and 1 PT (B. Tudzynski and coworkers, unpublished data). Up to date, only five secondary metabolites produced by *F. fujikuroi* could be assigned to a specific key enzyme. The polyketide pigments bikaverin and fusarubins are produced by the PKSs Bik1 (former Pks4) [21], [22] and Fsr1 [9], respectively, and Fus1 is the hybrid PKS/NRPS involved in fusarin formation (E.-M. Niehaus and B. Tudzynski, unpublished data). The bifunctional TC *ent*-copalyl diphosphate/*ent*-kaurene synthase (CPS/KS) is the key enzyme for *ent*-kaurene biosynthesis, the first step in GA formation [23], whereas CarRA is the TC involved in neurosporaxanthin production [24].

From a mechanistic point of view PKSs closely resemble fatty acid synthases (FASs). Similar to FASs, these multidomain enzymes contain acyl-carrier-proteins (ACPs) that covalently bind the growing acyl chain during PKS assembly. For functionality the ACP domains need to be post-translationally modified by 4′-phosphopantetheinyl (4′PPT) transferases (PPTases). These Mg^2+^-dependent enzymes catalyze the covalent linkage of the 4′PPT moiety of coenzyme A to a conserved serine residue within the ACP domains, where this 4′PPT linker functions as the carrier for the growing acyl chain. Similarly, NRPSs contain peptidyl-carrier-proteins (PCPs) for binding of the growing peptidyl chain that are also modified by 4′-phosphopantetheinylation of a conserved serine residue [25], [26]. In contrast to this post-translational modification of PKSs and NRPSs, TCs and PTs do not harbor a prosthetic group that is essential for full functionality.

In the yeast *Saccharomyces cerevisiae*, three PPTases have been identified. One is an integral part of the cytoplasmic type I FAS alpha-subunit (Fas2p) providing intrinsic catalytic activity only [27]. The second PPTase, Ppt2p, belongs to the AcpS-type PPTases and exclusively activates the low molecular weight ACP of the mitochondrial type II FAS [28]. The third PPTase, Lys5p, is a member of the Sfp-type PPTases and is essentially involved in lysine biosynthesis where it transfers 4′PPT to the α-aminoadipate reductase Lys2p [29]. In filamentous fungi homologs to all three yeast PPTases have been identified. Apart from the integral domain of the cytoplasmic FAS alpha-subunit PPTase, the PPTase PptB required for targeting the mitochondrial ACP (AcpA) was recently characterized in *Aspergillus fumigatus* [30]. The first homolog of the *S. cerevisiae* Sfp-type PPTase has been described in *A. nidulans* by two independent research groups who identified the genes responsible for the “null pigmented” and “cross-feedable white” phenotype of mutants, respectively. The gene loci were designated *npgA* and *cfwA*, respectively [31], [32]. Later both loci were identified to be identical and encode a Sfp-type PPTase [33], [34] that is responsible for penicillin, siderophore (extracellular triacetyl fusarinine C and intracellular ferricrocin), emericellin, shamixanthone, dehydroaustinol, and lysine production [33]–[35]. Other examples for altered secondary metabolite spectra were found in *npgA*/*ppt1* mutants of *Colletotrichum graminicola* [36], *Penicillium chrysogenum* [37], *A. niger* [38] and *Trichoderma virens* [39]. Furthermore, in *A. fumigatus*, the homologous PptA was shown to pantetheinylate the NRPS Pes1 [40]. Similarly to the *npgA/ppt1* mutant of *A. nidulans*, deletion mutants of the homologous genes in *C. graminicola*, *Magnaporthe oryzae* [36], *P. chrysogenum* [37], *A. fumigatus* [40], *A. niger* [38], *Cochliobulus sativus* [41] and *T. virens* [39] are lysine auxotrophic. Recent studies of the cereal pathogens *C. graminicola* and *C. sativus* have shown that Ppt1 is required for establishment of full virulence on rice and barley leaves, respectively. Addition of lysine did not restore wild-type-like virulence indicating the involvement of PKS- and/or NRPS-derived products in necrotrophic growth [36], [41]. Interestingly, *ppt1* mutants of *T. virens* are not affected in root colonization but cause attenuation of specific plant defense responses and hence an attenuated resistance against the fungal pathogen *Botrytis cinerea* [39].

Apart from its lysine auxotrophy the *A. nidulans npgA* mutant was unable to grow without the addition of NRPS-derived siderophores [35]. This dependency on siderophore-mediated iron uptake was not reported in any other species lacking the respective Sfp-type PPTase, most likely due to the existence of alternative reductive iron uptake systems. These alternative uptake systems are represented by ferroxidases and iron permeases that are missing in *A. nidulans* [42]. In *A. fumigatus* the only reductive iron uptake system which can be specifically inhibited by the iron chelator bathophenantroline disulfonate (BPS) is represented by the ferroxidase FetC and the iron permease FtrA that are arranged in a small cluster sharing one promoter [43]. From seminal work in *A. fumigatus* it is known that several genes that are involved in iron homeostasis (including *fetC* and *ftrA*) are controlled by a complex regulatory network that centers around the GATA-type transcription factor SreA [42]–[45]. In other fungal species iron-dependent regulation also involves SreA homologs called Urbs1 in *Ustilago maydis* [46], [47], SreP in *P. chrysogenum* [48] and Sre1 in *Histoplasma capsulatum* [49]. In *F. graminearum*, additionally to Nps6 which is the NRPS responsible for production of the extracellular siderophore [50] two ferroxidases (Fet1 and Fet2) and two iron permeases (Ftr1 and Ftr2) were recently identified to be involved in iron acquisition [51]. It was shown that Fet1 and Ftr1 are associated within the plasma membrane, whereas Fet2 and Ftr2 reside in the vacuolar membrane [51]. Whether transcriptional control of the encoding genes is mediated by a SreA homolog is yet unclear.

In this work, we report on the characterization of the first Sfp-type PPTase mutant generated in a species of the genus *Fusarium*, i.e. the rice pathogen *F. fujikuroi*. The work focuses on general growth characteristics regarding the dependency of the deletion mutant on lysine and iron, as well as developmental features (asexual and sexual differentiation) and pathogenicity on rice plants. Of special interest was the comparison of secondary metabolite profiles of the wild type and the Ff*ppt1* mutant regarding the ability to produce PKS and PKS/NRPS-derived *versus* terpene-derived products. Furthermore, we show that the deletion of Ff*ppt1* affects not only the biosynthesis of the PKS-, PKS/NRPS- and terpene-derived secondary metabolites but also the expression of genes coding for the respective key enzymes. Comparison of Ff*ppt1* deletion mutants in different *F. fujikuroi* strains with their respective wild-type strains points to a distinctive role of PKS and/or NRPS-derived products during sexual and asexual development. The ability of the Ff*ppt1* mutants to grow on iron deficient media led us to investigate the reductive iron uptake systems of *F. fujikuroi* including their transcriptional regulation. Additionally, pathogenicity assays on rice roots with fluorescently labeled Ff*ppt1* mutant and wild-type strains provide new insights into the role secondary metabolites play during the pathogen-host interaction.

## Results

### Identification and characterization of the Sfp-type PPTase gene Ffppt1 reveals involvement in lysine biosynthesis

**Figure 2:**
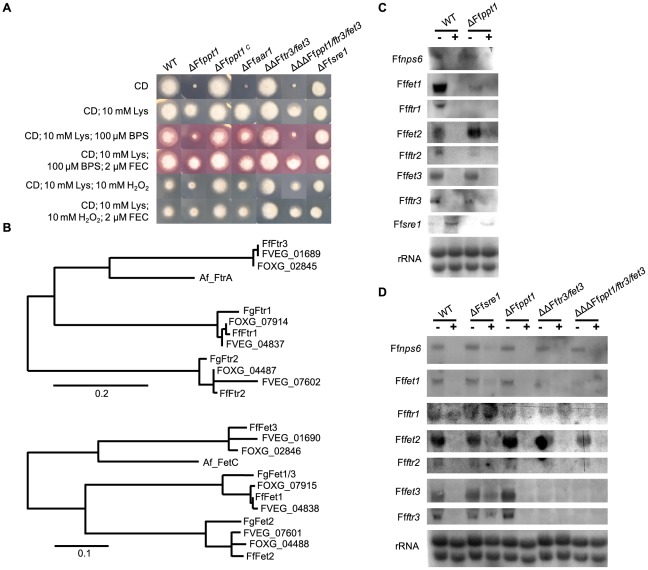
Influence of FfPpt1, FfSre1 and FfFtr3/FfFet3 on lysine biosynthesis, iron homeostasis and oxidative stress. A: Growth ability of the indicated strains on solidified Czapek Dox (CD) medium supplemented as indicated. Representative pictures were taken after 3 days of incubation at 28°C in the dark. B: Phylograms of ferroxidases and iron permeases from *F. fujikuroi* (Ff), characterized proteins from *F. graminearum* (Fg) and *A. fumigatus* (Af), as well as homologous sequences from *F. oxysporum* (FOXG) and *F. verticillioides* (FVEG) obtained from the Broad Institute database were created as described in Methods. Scale bars represent character changes. C and D: Northern blot analysis using indicated genes as probes and rRNA visualization as loading control. The indicated strains were grown as described in Methods. (−); addition of water, (+) addition of FeCl_3_ to a final concentration of 1 mm.

In order to identify the NpgA/CfwA-encoding homolog in the *F. fujikuroi* genome, a BlastP analysis was performed using the *A. fumigatus* PptA sequence. One protein sequence with 32% identity to PptA (e value = 2.6 e^−28^) was found and designated FfPpt1 (GenBank accession number HE614113). RT-PCR revealed an open reading frame of 876 bp spanning one intron of 50 bp. Targeted gene replacement using a nourseothricin resistance cassette yielded three transformants (designated ΔFf*ppt1* T8, T13, and T14). The loss of the Ff*ppt1* ORF was verified by diagnostic PCR (Fig. S1), and additional integrations of the resistance cassette into the genome were excluded by Southern blot (Fig. S1). In contrast to the wild type, the mutants were unable to grow on minimal medium without addition of lysine (Fig. 2A). Since all transformants exhibited the same phenotype, ΔFf*ppt1* T8 was arbitrarily chosen for further experiments. This mutant was complemented by re-integration of the Ff*ppt1* wild-type gene copy into the genome. Minimal medium without lysine was used for selection, and PCR verified re-integration of Ff*ppt1* for the transformants designated ΔFf*ppt1^C^* (Fig. S1).

To determine which of the pleiotropic defects of the ΔFf*ppt1* mutant are due to the non-functional α-aminoadipate reductase resulting in lysine auxotrophy, we performed a targeted deletion of the α-aminoadipate reductase gene Ff*aar1* (GenBank accession number HE614114). The deduced protein showed 49% identity to Aar1 from *C. graminicola* by BlastP analysis (e value = 0). Three out of 20 obtained transformants (ΔFf*aar1* T13, T14 and T20) proved loss of the target ORF by diagnostic PCR (Fig. S2A). The ΔFf*aar1* T13 mutant was used as a control for all further analyses.

Growth tests with the ΔFf*ppt1*, ΔFf*ppt1^C^*, and ΔFf*aar1* strains and the wild type were carried out on minimal medium containing either no lysine or 10 mm lysine. The ΔFf*ppt1^C^* strain showed wild-type-like growth on all media, whereas the ΔFf*aar1* and ΔFf*ppt1* strains did not grow on medium without lysine (Fig. 2A).

### FfPpt1 contributes to a functional iron uptake system that is controlled by the GATA-type transcription factor FfSre1

In addition to the growth defect on lysine-deficient medium, growth of the ΔFf*ppt1* strain was severely restricted on medium containing lysine in the presence of the iron chelator BPS and H_2_O_2_, respectively (Fig. 2A). This restriction was overcome when 2 µm of the siderophore ferrichrome (FEC) was added to the medium containing BPS/lysine and H_2_O_2_/lysine, respectively (Fig. 2A). These data suggest that siderophore-assisted iron uptake allows the *F. fujikuroi* wild type to grow in the presence of BPS, and that loss of siderophore production affects sensitivity against H_2_O_2_ in the *ppt1* mutant. The ability of ΔFf*ppt1* to grow on medium containing lysine and only trace amounts of iron (CD minimal medium contains 3.6 µm FeSO_4_) without the addition of siderophores suggested that a reductive iron uptake system is present in *F. fujikuroi*. BlastP analysis using the sequence of the plasma-membrane-localized Fet1 of *F. graminearum* against the genome database of *F. fujikuroi* revealed three proteins designated FfFet1 (89% identity, e-value = 0), FfFet2 (66% identity, e-value = 0), and FfFet3 (56% identity, e-value = 0). All three *F. fujikuroi* ferroxidase encoding genes revealed the presence of an adjacent iron permease encoding gene (designated Ff*ftr1*, Ff*ftr2*, and Ff*ftr3*, respectively) each sharing its promoter region with the adjacent Fet-encoding gene. Searching the available *Fusarium* genome sequences revealed that *F. verticillioides* and *F. oxysporum* each possess three of these pairs similar to *F. fujikuroi*. Phylogenetic analysis showed that the *A. fumigatus* FtrA/FetC cluster groups together with FfFtr3/FfFet3, but no respective homologs seem to be present in the *F. graminearum* genome (Fig. 2B). To test whether the respective genes are expressed in an iron-dependent manner, we grew the wild type and the Ff*ppt1* mutant under iron deficient conditions before addition of ferric chloride (FeCl_3_) or water. Northern analyses revealed that all of the genes coding for putative *F. fujikuroi* ferroxidases and iron permeases are induced by iron starvation in the wild type and the Ff*ppt1* mutant (Fig. 2C and D). Furthermore, the expression of the *F. fujikuroi* gene Ff*nps6* coding for the homolog of the *F. graminearum* NRPS Nps6 responsible for extracellular siderophore production [50] revealed an identical expression pattern (Fig. 2C and D). Expression signals of the SreA-encoding homologous gene Ff*sre1* in the wild type and the Ff*ppt1* mutant were found under iron excess only (Fig. 2C).

Since in *F. graminearum* the proteins Ftr1/Fet1 were shown to constitute a *bona fide* plasma membrane iron uptake system [51], [52] and Ftr2/Fet2 were suggested to function as a vacuolar iron transport system due to their localization [51], we wanted to investigate the role of newly identified FfFtr3/FfFet3 with respect to iron acquisition in the wild type and the siderophore-deficient Ff*ppt1* mutant. Targeted gene replacement of FfFtr3/FfFet3 was performed in both the wild-type strain and the ΔFf*ppt1* strain. Diagnostic PCR revealed three of eleven transformants to have lost the ORFs of both genes, respectively (Fig. S2B). These transformants were designated ΔΔFf*ftr3/fet3* (T3, T6 and T7) and ΔΔΔFf*ppt1/ftr3/fet3* (T1, T2 and T3) of which ΔΔFf*ftr3/fet3* T3 and ΔΔΔFf*ppt1/ftr3/fet3* T1 were arbitrarily chosen for further investigation. To learn more about the regulation of the genes putatively involved in iron acquisition in *F. fujikuroi* we also deleted the gene Ff*sre1*, encoding the homolog of the the GATA-type transcription factor Sre1 from *A. fumigatus*. This transcription factor was shown to be involved in regulation of iron homeostasis [44]. Of the eleven transformants obtained, seven were proven to have lost the ORF by diagnostic PCR (Fig. S2C) and designated ΔFf*sre1* (T1, T2, T3, T4, T6, T10, T11) of which T1 was arbitrarily chosen for further experiments.

As expected, the Ff*ppt1*/*ftr3*/*fet3* triple mutant was unable to grow without the addition of lysine and showed restricted growth in the presence of H_2_O_2_ (Fig. 2A). Interestingly, the Ff*ftr3*/*fet3* double and the Ff*ppt1*/*ftr3*/*fet3* triple mutant exhibited a slightly less restricted growth in the presence of H_2_O_2_ compared to the wild type and the Ff*ppt1* single mutant, respectively (Fig. 2A). When additional 2 µm of the siderophore FEC was present, the growth defect of the Ff*ppt1* single mutant could partially be overcome whereas the Ff*ftr3*/*fet3* double and the Ff*ppt1/ftr3*/*fet3* triple mutant were restored to wild-type-like growth (Fig. 2A). In contrast, in the presence of BPS the Ff*ppt1*/*ftr3*/*fet3* triple mutant showed a more severe growth defect compared to the Ff*ppt1* single mutant (Fig. 2A). The overall picture of growth ability of the mutants on the different media indicates that FfPpt1, FfFtr3 and FfFet3 participate in iron acquisition and mediate H_2_O_2_ tolerance in *F. fujikuroi*. The Ff*sre1* deletion mutant showed restricted growth compared to the wild type on all media tested (Fig. 2A) and no growth when 1 mm FeCl_3_ was present (Fig. S3). To learn more about the role FfSre1 plays in regulation of genes involved in iron metabolism and to investigate whether transcriptional deregulation can be observed when the genes encoding FfFtr3/FfFet3 are missing, we performed northern blot analyses of the wild type and the ΔΔΔFf*ppt1/ftr3/fet3*, ΔFf*ppt1*, ΔFf*sre1* and ΔΔFf*ftr3/fet3* strains. Similarly to the observations from the previous iron-shift experiment, signals of Ff*nps6*, Ff*ftr1*, Ff*fet1*, Ff*ftr2* and Ff*fet2*, were only visible under nitrogen starvation conditions in the Ff*ppt1/ftr3/fet3* and Ff*ftr3/Fffet3* mutants and the parental strains (Fig. 2D). The fact that signals for Ff*nps6*, Ff*ftr1*, Ff*fet1*, Ff*ftr2* Ff*fet2*, Ff*fet3* and Ff*ftr3* were detectable in the Ff*sre1* mutant even when 1 mm FeCl_3_ was present (Fig. 2D) indicates that FfSre1 acts as a repressor of genes involved in iron metabolism in *F. fujikuroi*.

### FfPpt1 is involved in conidiogenesis and sexual development

**Figure 3:**
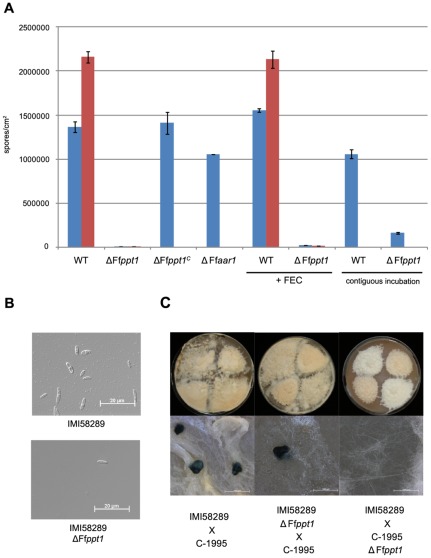
Influence of FfPpt1 on conidiogenesis and sexual mating recognition. A: Spores produced of indicated strains per cm^2^ after 10 days of incubation on solidified V8 medium in constant light conditions. Blue: strain IMI58289; red: strain C-1995. FEC: medium was supplemented with 2 µm ferrichrome; contiguous incubation: strains were incubated as described in Methods. B: DIC images of spores produced by the indicated strain. Strains were incubated and spores were collected as described in Methods. C: Representative photographs of sexual crossings of indicated strains as described in Methods.

*A. nidulans npgA/cfwA* mutants showed delayed and reduced spore formation with an altered morphology at a range of 32°C to 37°C, but could be restored when contiguously grown to the wild type [34]. Since deletion mutants of the respective homologs in several filamentous fungi were also affected in conidiogenesis or conidia morphology, we investigated the effect of the Ff*ppt1* deletion in *F. fujikuroi*. As to our knowledge none of the previous studies on PPTase mutants in any filamentous fungus compared sporulation ability to that of an α-aminoadipate reductase mutant, we included the Ff*aar1* mutant in our analysis. Similarly to the observations in other fungi, sporulation was severely reduced, but morphologically unaltered, in the Ff*ppt1* mutant compared to the wild type, the ΔFf*ppt1^C^* and ΔFf*aar1* strains (Fig. 3A and B). However, when the Ff*ppt1* mutant was grown contiguously to the wild type separated by water-permeable cellophane membrane sporulation was partially restored (Fig. 3A). Interestingly, this partial restoration was not observed when the individually cultivated Ff*ppt1* mutant was supplemented with FEC indicating that iron limitation is not responsible for the sporulation defect (Fig. 3A).

To study the influence of FfPpt1 on sexual development, a gene replacement mutant of the Ff*ppt1* locus was generated in the *F. fujikuroi* strain C-1995 that carries the *MAT1-2* idiomorph using the same targeted deletion strategy. Similarly to the Ff*ppt1* mutant in the wild-type strain IMI58289, the deletion mutant in C-1995 was lysine auxotrophic (Fig. S4) and exhibited drastically reduced sporulation ability (Fig. 3A). When performing sexual crosses, the wild-type strain IMI58289 and all mutant strains generated in this background (ΔFf*ppt1*, ΔFf*ppt1^C^* and ΔFf*aar1*) carrying the *MAT1-1* idiomorph were able to interact with the wild-type strain C-1995 resulting in the formation of dark purple perithecia (Fig. 3C and S5). However, when the Ff*ppt1* locus was missing in strain C-1995 carrying the *MAT1-2* idiomorph, no recognition took place when contiguously grown with any of the strains of the opposite mating type (wild-type IMI58289, ΔFf*ppt1*, ΔFf*ppt1^C^* and ΔFf*aar1*). Subsequently, colonies from both mating partners did not come in close contact resulting in a lack of perithecia (Fig. 3C and S5). Supplementation with FEC did not restore formation of sexual structures (Fig. S5).

### Deletion of Ffppt1 results in loss of PKS- and PKS/NRPS-derived products and in transcriptional down-regulation of distinct secondary metabolite cluster genes

**Figure 4:**
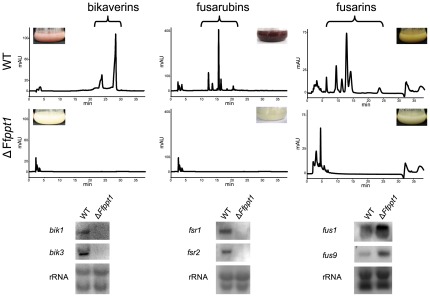
Involvement of FfPpt1 in PKS- and PKS/NRPS-derived secondary metabolite production and gene regulation. HPLC-UV chromatograms (bikaverins (510 nm) fusarubins (450 nm) and fusarins (363 nm)) in relative units (mAU) of indicated strains incubated as described in Methods. For HPLC conditions see Methods. Northern blot analyses of indicated strains from the same culture conditions probed with indicated cluster genes and rRNA visualization as loading control.

Since deletion of Sfp-type PPTase-encoding genes in several fungal organisms resulted in absence of PKS-, NRPS- and PKS/NRPS-derived metabolites, we assessed production of *F. fujikuroi* metabolites in the Ff*ppt1* mutant. The wild type and the Ff*ppt1* mutant were cultivated under bikaverin-, fusarubin-, and fusarin-stimulating conditions, respectively. The wild type exhibited the typical coloration for each of the three metabolites in the respective induction medium, whereas the Ff*ppt1* mutant appeared colorless in all three experiments (Fig. 4). Analysis of the culture filtrates using high performance liquid chromatography (HPLC) coupled to an diode array detector (DAD) for bikaverin and fusarubins and to an ultraviolet light (UV) detector for fusarin detection, respectively, confirmed the absence of bikaverins, fusarubins and fusarins in the Ff*ppt1* mutant in contrast to the wild type (Fig. 4). Surprisingly, northern blot analyses revealed that genes encoding the PKS key enzymes for bikaverin and fusarubin production, respectively, are negatively affected on transcriptional level when Ff*ppt1* is deleted (Fig. 4). Furthermore, other bikaverin and fusarubin cluster genes are affected in the same manner in the Ff*ppt1* mutant in contrast to the wild type, the Ff*aar1* and the Ff*ppt1* add-back strains (Fig. 4 and S6A). As expected, deletion of Ff*ppt1* does not affect the expression of all PKS- or NRPS-encoding genes tested: expression of *fus1*, encoding the PKS/NRPS hybrid responsible for fusarin production, was not repressed in the Ff*ppt1* mutant although no fusarins could be detected in the culture filtrate (Fig. 4). Similarly, expression of the NRPS-encoding Ff*nps6* and PKS-encoding Ff*pks6* genes was not effected in the Ff*ppt1* mutant compared to the wild type (Fig. 2 and Fig. S6B).

Some *F. fujikuroi* strains are able to produce the mycotoxin moniliformin, which was assumed to be a PKS-derived metabolite [53]. To test this hypothesis we deleted the *ppt1* locus in the highly moniliformin-producing wild type strain MRC2276. Analyses using HPLC coupled to Fourier transformation mass spectrometry (FTMS) showed that the wild-type strain as well as the Ff*ppt1* deletion mutant was able to form moniliformin in detectable amounts (Fig. S6C), suggesting that no Sfp-type PPTase activity is required for moniliformin production.

### Influence of FfPpt1 on the production of sesqui- and diterpenes

**Figure 5:**
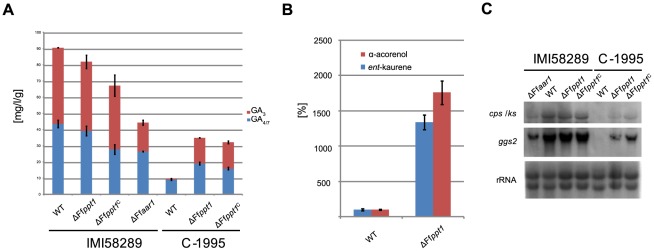
Effect of Ff*ppt1* deletion on terpene-derived metabolites. A: HPLC quantified amounts of GA_3_ (red) and the sum of GA_4_ and GA_7_ (blue) in mg per l culture and mycelium dry weight in mg of indicated strains. Data are given as means and standard deviations of two biological replicates. For cultivation and HPLC conditions see Methods. B: Quantified amounts of α-acorenol (red) and *ent*-kaurene (blue) of indicated C-1995 strains by GC-MS. Data are given as means and standard deviations of three biological replicates. For cultivation and GC-MS conditions see Methods. C: Northern blot analysis of the first GA cluster genes of the indicated strains. rRNA visualization as loading control.

To show if the loss of PKS-and NRPS-derived products in the Ff*ppt1* deletion mutants have an effect on biosynthesis of terpenes, strains IMI58289 (GA high-producing) and C-1995 (GA low-producing) were investigated for their production of diterpenoid GAs, the GA precursor *ent*-kaurene and the sesquiterpene alcohol α-acorenol. The latter was recently identified as the main volatile sesquiterpene produced by *F. fujikuroi* [8]. Quantification of GA in culture extracts by HPLC-DAD revealed no significant increase of GAs in the IMI58289/ΔFf*ppt1* mutant compared to IMI58289 (Fig. 5A), while deletion of Ff*ppt1* in the strain C-1995 resulted in a dramatic increase of GA production compared to the parental strain C-1995. Accordingly, GC-MS analysis of headspace extracts obtained by use of a closed loop stripping apparatus (CLSA) demonstrated that deletion of Ff*ppt1* in strain C-1995 led to a significant increase in *ent*-kaurene and α-acorenol production (Fig. 5B). These findings on the secondary metabolite level coincided with the expression levels for the genes *ggs2* and *cps*/*ks* encoding the first two enzymes of GA biosynthesis: the signals were dramatically increased in the Ff*ppt1* mutant of strain C-1995 compared to the wild type, but only marginally altered in the IMI58289 Ff*ppt1* mutant compared to its parental strain (Fig. 5C). Surprisingly, a significantly decreased production of GAs was obtained in the Ff*aar1* deletion mutant. In full agreement with this finding, expression signals for *cps*/*ks* and *ggs2* were also significantly reduced compared to the Ff*ppt1* mutant and the wild-type IMI58289 (Fig. 5C).

### FfPpt1 is a pathogenicity factor in hydroponic rice cultures

**Figure 6:**
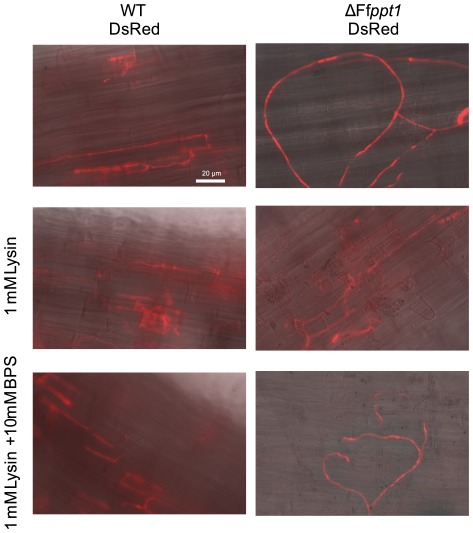
Fluorescence microscopy of Ffppt1 and wild-type strains during rice root infection assays. Representative fluorescent microscopy pictures of indicated strains in rice root infection assays performed as described in Methods. Gamborg B5 Medium was supplemented as indicated.

To investigate whether the loss of PKS- and NRPS-derived compounds by deleting Ff*ppt1* affects rice root infections, the wild-type strain and the Ff*ppt1* mutant were transformed with the vector pHphDsRed conveying constitutive expression of the red fluorescent protein (DsRed). The DsRed-tagged wild type and Ff*ppt1* mutant were inoculated onto roots of germinated rice seedlings in the presence or absence of lysine and lysine plus BPS, respectively. Fluorescence microscopy showed that the wild type was able to penetrate and infect rice root cells under all conditions tested (Fig. 6). The Ff*ppt1* mutant established infection patterns in the presence of lysine only. No interaction of the Ff*ppt1* mutant with the rice roots was observed in the absence of lysine or the presence of lysine when BPS was supplemented concurrently. The hyphae show a non-oriented growth on the root surface and do not penetrate (Fig. 6).

## Discussion

### FfPpt1 is essential for lysine biosynthesis and involved in iron acquisition

In *S. cerevisiae* it was proven that the Sfp-type PPTase Lys5p is essential for activating the apo α-aminoadipate reductase Lys2p by 4′phosphopantetheinylation and therefore the enzyme is essentially involved in lysine biosynthesis [29]. As expected, targeted deletion of the homologous gene *ppt1* in *F. fujikuroi* resulted in lysine auxotrophic mutants demonstrating that Ff*ppt1* is essential for lysine biosynthesis. The high degree of amino acid conservation of FfPpt1 to Lys5p makes it very likely that FfPpt1 activates the *F. fujikuroi* α-aminoadipate reductase Aar1 in the same mode of action as Lys5p activates Lys2p. The involvement of FfPpt1 in lysine biosynthesis is consistent with the observations made in several filamentous ascomycetes [30], [33], [36]–[39], [41]. Since Ff*ppt1* mutants were not viable without lysine supplementation it is suggested that FfAar1 cannot be post-translationally modified by the mitochondrial AcpS-type PPTase homolog.

*A. nidulans npgA/cfwA* mutants exhibit an iron uptake deficiency additional to a lysine auxotrophy since the production of NRPS-derived intra- and extra-cellular siderophores is abrogated and no additional reductive iron uptake system is present [35]. Similarly, the production of the NRPS-derived siderophores of *C. graminicola* is also dependent on Sfp-type PPTase activity, but respective PPTase mutants are able to grow without siderophore addition indicating the existence of a reductive iron uptake system [36]. In fact, absence of reductive iron acquisition systems has not been reported for any other fungal species other than *A. nidulans* [42]. Accordingly, the Ff*ppt1* mutants were able to grow without the addition of siderophores in the presence of lysine indicating the existence of such reductive iron uptake system also in *F. fujikuroi*. Furthermore, the ability of the *F. fujikuroi* wild type strain to grow in the presence of the iron chelator BPS, which specifically inhibits the reductive iron uptake system, strongly indicates the existence of NRPS-derived siderophores in *F. fujikuroi*. Since in the closely related species *F. graminearum* two NRPS-encoding genes were recently shown to be responsible for production of the extracellular siderophore triacetyl fusarine C [50] and the intra-cellular siderophore ferricrocin [54], respectively, the homologous genes in *F. fujikuroi*, Ff*nps6* and Ff*nps2*, are very likely required for siderophore production. However, the nature of the *F. fujikuroi* siderophores has to be elucidated in future experiments. Nevertheless, the involvement of FfPpt1 in siderophore biosynthesis is evident since respective mutants were severely restricted in growth when grown in the presence of BPS and lysine, resembling the situation in *A. nidulans* and *C. graminicola* [35], [36]. This growth defect could be restored when FEC, which functions as intra- and extra-cellular siderophore in *Schizosaccharomyces pombe* [55], was supplemented, suggesting that it can be utilized by *F. fujikuroi*. Similar observations of FEC utilization were made in *A. nidulans* [56].

### Ftr3/Fet3 are involved in iron metabolism of *F. fujikuroi*

Since Ff*ppt1* mutants were able to grow without siderophore addition in the presence of lysine but not when additional BPS was present, the existence of reductive iron uptake systems was suggested in *F. fujikuroi*. In *F. graminearum* two reductive iron uptake systems were identified, each consisting of a ferroxidase and an iron permease [51], [52]. Blast searches performed in the recently obtained *F. fujikuroi* genome database revealed the existence of three gene pairs each encoding an iron permease and a ferroxidase that share one promoter region. Phylogenetic analysis shows that two of them, FfFtr1/FfFet1 and FfFtr2/FfFet2, are closely related to the characterized proteins from *F. graminearum*, and that the newly identified proteins FfFtr3 and FfFet3 group together with FtrA and FetC from *A. fumigatus*, respectively. Similarly to the *ftrA* deletion mutant of *A. fumigatus* [43], *F. fujikuroi* Ff*ftr3*/Ff*fet3* deletion mutants were not restricted in growth compared to the wild type, most likely due to the production of siderophores. However, when Ff*ftr3*/Ff*fet3* were deleted in a Ff*ppt1* mutant background the growth defect in the presence of BPS was more dramatic, indicating that FfFtr3/FfFet3 are involved in iron acquisition but can be complemented by another iron uptake system. Generation of triple mutants of Ff*ppt1* and Ff*ftr3*/Ff*fet3* together with either Ff*ftr1*/Ff*fet1* or Ff*ftr2*/Ff*fet2* could resolve the question if the identified putative reductive iron uptake systems have redundant functions. Furthermore, it would be interesting to investigate if FfFtr3/FfFet3, which are not present in *F. graminearum*, could restore iron transport in Fg*ftr1*/Fg*fet1* mutants.

### FfSre1 controls iron metabolism

Similarly to the expression of *ftrA* and *fetC* observed in *A. fumigatus* [44] and genes involved in reductive iron uptake in *H. capsulatum* [49], all of the six genes putatively involved in reductive iron uptake in *F. fujikuroi* were shown to be expressed under iron deficient conditions and repressed upon iron addition, strongly suggesting their role in iron metabolism. Furthermore, expression of Ff*nps6* encoding the homolog of the *F. graminearum* NRPS, responsible for extracellular siderophore production [50], revealed the same expression pattern thereby indicating a role of FfNps6 in iron homeostasis. The homolog in *A. fumigatus*, *sidD*, was also shown to be repressed by the addition of iron [44] underlining our hypothesis. In *A. fumigatus* and *H. capsulatum* the GATA-type transcription factor-encoding genes *sreA* and *sre1*, respectively, are expressed under iron sufficiency and act as repressors of genes involved in iron acquisition [44], [49]. The *F. fujikuroi* homologous gene Ff*sre1* is also expressed under iron sufficient conditions. Deletion resulted in deregulation of all six genes putatively involved in reductive iron uptake as well as Ff*nps6* when iron was supplemented, indicating that FfSre1 executes comparable repressing functions as SreA/Sre1 in *A. nidulans*, *A. fumigatus* and *H. capsulatum*, respectively [44], [49], [57], [58]. Interestingly, Ff*sre1* deletion mutants were not viable in the presence of constant iron excess, which is likely due to a lack of repression, leading to increased iron flux into the cells. The increased intracellular iron pool might function in Fenton/Harber Weiss chemistry generating oxidative stress to a toxic level. However, under physiological iron concentrations Ff*sre1* mutants exhibited a decreased sensitivity to H_2_O_2_ compared to the wild type, which might either be due to an increased intracellular siderophore concentration possibly scavenging free iron from Fenton/Harber Weiss chemistry and/or by specifically providing more intracellular iron as cofactor for the iron-dependent catalase known to detoxify H_2_O_2_. Supporting this hypothesis, addition of FEC to Ff*ppt1* mutants increases H_2_O_2_ resistance. This hypothesis is consistent with findings in *A. nidulans* and *A. fumigatus* where loss of intracellular siderophores increases the liable intracellular iron pool leading to a decreased oxidative stress resistance [59], [60]. Future studies focusing on the involvement of FfSre1 in iron metabolism and oxidative stress responses in *Fusarium* species will help to elucidate this complex context. Here, investigating the role of the homolog of the bZIP transcription factor HapX will be of special interest. In *A. fumigatus* HapX was shown to be a transcriptional repressor of genes involved in iron consuming pathways and an activator of genes involved in iron acquisition under iron deficiency. The HapX encoding gene itself is transcriptionally repressed by SreA under iron replete conditions [61].

### Ppt1 controls secondary metabolism in *F. fujikuroi*

The findings that putatively NRPS-derived siderophore biosynthesis is affected in Ff*ppt1* mutants led us to investigate a broader PKS- and PKS/NRPS-derived secondary metabolite spectrum of *F. fujikuroi*. Similarly to the findings in several filamentous ascomycetes [33]–[39], *F. fujikuroi ppt1* mutants were not able to produce any PKS-derived products such as bikaverins, fusarubins or PKS/NRPS-derived fusarins. This is in agreement with the fact that Sfp-type PPTases are essential for 4′-phosphopantetheinylation of ACPs and PCPs in PKSs and NRPSs, respectively [25], [26]. However, production of moniliformin, that was suggested to be produced in a PKS-dependent manner [53], was not altered in Ff*ppt1* mutants, indicating a biosynthetic pathway that is independent of Sfp-type PPTase activity in *F. fujikuroi*. Production of TC-derived secondary metabolites such as the diterpenoid GAs and the sesquiterpene alcohol α-acorenol was not negatively affected when *ppt1* was deleted in *F. fujikuroi*. However, when the α-aminoadipate reductase-encoding gene Ff*aar1* was deleted a significant reduction of GAs could be observed. A similar negative effect on secondary metabolism was found when the glutamine synthetase-encoding gene *glnA* was deleted in *F. fujikuroi* [62]. How FfAar1 is involved in GA metabolism remains elusive and awaits clarification in the future.

The effect of *ppt1* deletion on GA and α-acorenol production in *F. fujikuroi* was shown to be strain-dependent. The *ppt1* knock-out in strain C-1995 that produces very low amounts of GAs resulted in a significant increase in GA and α-acorenol production (ca. 15-fold increase), while the deletion in the highly GA-producing strain IMI58289 had almost no effect. However, since the Ff*aar1* mutant in IMI58289 resulted in a significant decrease in GA production, but the Ff*ppt1* mutant produced wild-type-like amounts although FfAar1 is not functional, an increase in GA production can be observed that might be restricted to a wild-type level due to saturated enzyme activity in downstream reactions of the biosynthetic pathway in IMI58289. In summary, we postulate that the increased flux of acetyl-CoA precursor molecules is channeled into the terpene metabolism due to a block of PKS and NRPS pathways. A similar phenomenon of redirected secondary metabolite carbon flux occurs in *Taraxacum brevicorniculatum* when the *cis*-prenyltransferases responsible for natural rubber production are silenced [63].

An interesting observation is the specific transcriptional down-regulation for the two non-reducing PKS-encoding genes, *bik1* and *fsr1*, as well as additional genes from the corresponding clusters that was not observed for any other secondary metabolite key enzyme tested in Ff*ppt1* mutants. A similar effect has not been reported in any other filamentous fungi investigated for secondary metabolism in Sfp-type PPTas deficient mutants so far. Although the nature of this transcriptional effect cannot be resolved, it is supported by the findings that a transcriptional interdependency of the bikaverin and the fusarubin structural enzymes was observed in earlier studies [9], [22]. Future experiments will have to provide evidence whether this phenomenon is related to non-reducing PKS gene clusters in general or specific for the bikaverin and fusarubin gene clusters in *F. fujikuroi*.

### FfPpt1 is involved in asexual and sexual development most likely by inactivating PKS and/or NRPS pathways

We noted that Ff*ppt1* mutants revealed a significant reduction of conidiation that was independent from FEC supplementation, but could be restored when the mutant was grown contiguously to the wild-type strain. These data suggest that possible PKS- and/or NRPS-derived metabolites can function as diffusible conidiation signals in *F. fujikuroi*. In *A. nidulans*, sporulation is also suggested to be induced by a diffusible signal that involves the activity of the glutamine synthetase-like enzyme FluG [64]. The *npgA/cfwA* and *fluG* mutants showed a similar restoration of sporulation in contiguous growth experiments [34], [64]. Recently it was shown that the sporulation defect of *A. nidulans fluG* mutants could be rescued by the concomitant addition of specific TC- and PKS-derived products [65]. Further evidence for the existence of diffusible sporulation signals comes from *Ascochyta pisi*, where the metabolite P310/mycosporine was shown to induce sporulation [66], [67]. Mycosporines are produced by many fungal and marine organisms [68]. In cyanobacteria it was proven that this group of metabolites involves a NRPS during biosynthesis [69]. From the data obtained in this study it is intriguing to speculate that sporulation in *F. fujikuroi* also involves either a mycosporine-like NRPS product similar to *A. pisi* and/or a distinct mixture of secondary metabolites as reported in *A. nidulans*.

Apart from the defect in conidiation of *F. fujikuroi ppt1* mutants, we also observed a defect in formation of sexual structures when Ff*ppt1* was deleted in the *MAT1-2* mating partner and crossed with a wild-type *MAT1-1* strain, whereas the *vice versa* situation led to formation of dark purple perithecia. In several ascomycetes, two NRPS-independent peptide pheromone/receptor systems that underlie transcriptional control of the *MAT1-1* and *MAT1-2* idiomorphs are a prerequisite for mating recognition in heterothallic species [70]. Although the *MAT1-1* and *MAT1-2* idiomorphs as well as the genes encoding the pheromone/receptor systems have recently been identified in heterothallic *Fusarium* species including *F. fujikuroi* [71] an involvement in mating recognition awaits experimental proof. Interestingly, in the homothallic species *F. graminearum*, which contains both *MAT* idiomorphs, deletion of one idiomorph prevents self-fertilization but mutants maintain the ability to outcross [72]. However, recent studies in *F. graminearum* revealed that the genes coding for the pheromone/receptor systems are not essential for self-fertilization and outcrossing [73], [74]. Our results suggest that FfPpt1 is involved in sexual recognition, disclosing the possibility that the *MAT1-2* idiomorph specifically controls a PKS- and/or NRPS-derived metabolite or its receptor. And although differential cDNA screening and microarray analyses of *MAT1-2* deletion mutants of *F. verticillioides* and *F. graminearum* did not reveal any apparent Sfp-type PPTase target or putative receptor-encoding gene to be transcriptionally controlled by the *MAT1-2* idiomorph [75], [76], our hypothesis should thoroughly be investigated in *F. fujikuroi*.

### FfPpt1 is a pathogenicity factor during rice root infection of *F. fujikuroi*

The data obtained from pathogenicity assays of fluorescently labeled *F. fujikuroi ppt1* mutants in hydroponic rice cultures indicate that lysine production and iron uptake are essential for the establishment of infection of the rice roots. However, when lysine was supplemented, wild-type-like infection structures of the *ppt1* mutant could be observed, indicating that the reductive iron uptake systems of *F. fujikuroi* are sufficient for iron acquisition during infection. An essential role for reductive iron uptake was reported in the smut fungus *U. maydis* during maize infection [77], whereas *ftrA* mutants of *A. fumigatus* were not affected in virulence in a murine model of invasive aspergillosis [43]. Interestingly, in *F. graminearum*, which lacks the Fet3/Ftr3 homologous system, siderophore-assisted iron uptake is essential for full pathogenicity on wheat [52]. Our data further suggest that no PKS- and NRPS-derived metabolites of *F. fujikuroi* are essential for primary invasion of rice roots. This stands in contrast to the observations reported for Sfp-type PPTase mutants of the hemibiotrophic plant pathogens *C. graminicola* and *M. oryzae* that were unable to cause primary infections [36], and *C. sativus* that showed strongly reduced primary infections on unwounded host plant leaves, respectively [41]. However, *T. virens* Sfp-type PPTase mutants were able to colonize *Solanum lycopersicum* roots in a wild-type-like manner when lysine was supplemented [39]. The theory that plant pathogenic fungi and bacteria need PKS- and NRPS-derived secondary metabolites for establishment of full virulence is reported in several species [78]–[83]. Whether this is also true for *F. fujikuroi* needs to be investigated in a more sophisticated pathogenicity assay in the future. It is possible that the species-specific production of GAs in *F. fujikuroi* is the main determinant of host specificity and is essential for primary infection of rice roots. The fact that *F. fujikuroi* strains lacking the global regulator *velvet* are defective in both GA production and virulence support our suggestion on the role of TC-derived GAs for the infection of rice roots [84]. Examples for the essential role of other TC-derived secondary metabolites during infection come from the gray mold fungus *B. cinera* [85] and the more closely related species *F. graminearum* [86]. Analyses of the roles different *F. fujikuroi* secondary metabolites play during primary infection of rice will be a major task in the future.

Summarizing, our studies describe the first investigations centering on a Sfp-type PPTase in the genus *Fusarium*. We show unequivocally that FfPpt1 is essentially involved in primary metabolism of lysine biosynthesis and in PKS-, PKS/NRPS- and NRPS-derived secondary metabolites such as bikaverins, fusarubins, fusarins, and most likely siderophores. Surprisingly, moniliformin production was not affected indicating that the biosynthetic pathway does not involve a Sfp-tye PPTase-dependent PKS. Furthermore we provide strong evidence that deletion of Ff*ppt1* causes re-channelling of carbon flux into the terpene metabolism which results in increased GA and α-acorenol production. Therefore, Ff*ppt1* mutants provide a reasonable strain improvement strategy for terpene-derived secondary metabolite production. Pathogenicity assays using hydroponic rice cultures revealed that lysine biosynthesis and iron acquisition, but not PKS and NRPS secondary metabolism is essential for establishing primary infections of *F. fujikuroi* on rice roots. Additionally, the results obtained disclose the possibilities that PKS- and/or NRPS-derived metabolites might function as diffusible conidiation signals and also might play a crucial role in mating recognition in dependency on a specific *MAT* idiomorph in heterothallic *Fusarium* species.

Furthermore, our studies revealed the existence of a third previously unidentified putative reductive iron uptake system consisting of FfFtr3 and FfFet3 that is closely related to the only reductive system, FtrA/FetC, in *A. fumigatus*. Functional characterization using targeted deletion of both genes, Ff*ftr3*/Ff*fet3*, provides strong evidence that they are involved in iron acquisition and under transcriptional repression of the GATA-type transcription factor FfSre1 under iron-replete conditions. Deletion of the first *Fusarium* homolog of this GATA-type transcription factor strongly indicates its involvement in regulation of iron homeostasis and oxidative stress resistance, providing evidence for conserved regulation mechanisms between *Fusarium* and *Aspergillus* species in this respect.

## Methods

### Fungal strains and culture conditions

The wild-type strains *F. fujikuroi* IMI58289 (Commonwealth Mycological Institute, Kew, UK), *F. fujikuroi* C-1995 (kindly provided by J.F. Leslie, Kansas State University), and the moniliformin-producing strain *F. fujikuroi* MRC2276 (kindly provided by W. F. O. Marasas, Research Institute for Nutritional Diseases, South Africa) were used for *ppt1* knock-out experiments. For all cultures, *F. fujikuroi* was preincubated at 28°C for 48 h in 300 ml Erlenmeyer flasks with 100 ml Darken medium (DVK) [87] on a rotary shaker at 180 rpm. For RNA isolation and secondary metabolite analyses, 0.5 ml DVK were used to inoculate synthetic ICI (Imperial Chemical Industries Ltd., UK) media [88] containing either 6 mm glutamine (GAs and bikaverins), 60 mm glutamine (fusarins) or 6 mm NaNO_3_ (fusarubins). For cultivation including Ff*ppt1* mutants, lysine was added to all media to give a final concentration of 1 mm. The cultures were incubated at 28°C on a rotary shaker at 190 rpm for 3, 5, 7 or 10 days. For iron shift experiments FeCl_3_ was added to a final concentration of 1 mm and incubated for 2 h. For moniliformin analyses, the strains were grown at 28°C for 14 days on cracked corn as previously described [89]. Headspace analyses were performed with agar plate cultures grown on complete medium (CM) [90] amended with 1 mm lysine after three days of incubation. For protoplasting, 0.5 ml of the starter culture was transferred into Erlenmeyer flasks with 100 ml ICI medium containing 6 mm (NH_4_)_2_SO_4_ and 10 g/L fructose instead of sucrose and incubated at 28°C on a rotary shaker at 190 rpm for 18 h. For DNA extraction, fungal strains were grown for 3 days at 28°C on cellophane sheets (Alba Gewürze, Bielefeld, Germany) placed on solidified CM. For sporulation assays solidified 20% (v/v) vegetable juice (V8) (Campbell Foods, Puurs, Belgium) containing 30 mm CaCO_3_ and 10 mm lysine was used. Additionally solidified V8 contained 2 µm ferrichrome (Sigma-Aldrich Chemie GmbH, Steinheim, Germany) as indicated. After 10 days incubation under constant light spores were washed of the plates, filtered and counted using a hemocytometer. For growth tests 5% (w/v) Czapek Dox (CD) medium (Sigma-Aldrich Chemie GmbH, Steinheim, Germany) was used and complemented with 10 mm lysine, 100 µM BPS, 2 µM FEC as indicated. For additional growth test, solidified CM without iron was used containing 10 mm lysine and 1 mm FeCl_3_ when indicated. Sexual crossings were performed on carrot agar containing 10 mm lysine plus 2 µm FEC when indicated as described by Klittich and Leslie [91]. Microscopy of perithecia was performed using a SteREO Discovery.V20™ microscope equipped with an AxioCam MRc (Carl Zeiss MicroImaging GmbH, Jena, Germany). Spores were visualized using an Axio Imager.M2 (Carl Zeiss MicroImaging GmbH, Jena, Germany). Differential interference contrast (DIC) was used for bright field images captured with uniform exposure time using an AxioCam MRm. Images were processed uniformly using AxioVision Rel. 4.8 (Carl Zeiss MicroImagingGmbH, Jena, Germany).

### Standard molecular methods

DNA and RNA analysis used standard techniques [92]. Fungal DNA or RNA was prepared by first grinding lyophilized mycelium into a fine powder with a mortar and pestle and then dispersing it in extraction buffer as described by Cenis [93]. DNA for Southern hybridization experiments was prepared following the protocol of Doyle and Doyle [94]. For Southern blot analysis, genomic DNA was digested with the indicated restriction enzymes (Fermentas GmbH, St. Leon-Rot, Germany), fractionated in 1% (w/v) agarose gels, and transferred to Nytran® nylon transfer membranes (Whatman Inc., Sanford, ME, USA) by downward blotting [95]. ^32^P-labelled probes were prepared using the random oligomer-primer method and membranes were hybridized according to the protocol of Sambrook et al. [92].

Total *F. fujikuroi* RNA was isolated using the RNAgents total RNA isolation kit (Promega GmbH, Mannheim, Germany). Samples of 20 µg of total RNA were transferred to Hybond-N^+^ membranes after electrophoresis on a 1% (w/v) agarose gel containing 1% (v/v) formaldehyde, according to Sambrook et al. [92]. Northern blot hybridizations were accomplished by the method of Church and Gilbert [96]. cDNA was synthesized from 1 µg of total RNA and the SuperScript II reverse transcriptase (Invitrogen, Groningen, The Netherlands) according to the manufacturer's instructions.

All primers used for PCR were obtained from Eurofins GmbH (Ebersberg, Germany) (Table S1). PCR reactions contained 25 ng DNA, 5 pmol of each primer, 200 nm dNTPs, and 1 unit of BioTherm™DNA polymerase (GeneCraft GmbH, Lüdinghausen, Germany) and were initiated with a 4 min soak at 94°C followed by 36 cycles of 1 min at 94°C, 1 min at 56 to 65°C, 1–3 min at 70°C, and a final soak for 10 min at 70°C. PCR products were cloned into pCR®2.1-TOPO® vector using the TOPO TA Cloning® kit (Invitrogen, Groningen, The Netherlands) and transformed into *Escherichia coli* (Invitrogen). Plasmid DNA from *E. coli* was extracted using the GeneJET™ Plasmid Miniprep Kit (Fermentas GmbH, St. Leon-Rot, Germany) and sequenced using the BigDye® Terminator v3.1 Cycle Sequencing Kit and the ABI Prism® 3730 Genetic Analyzer (Applied Biosystems, Foster City, CA, USA) according the manufacturer's instructions. DNA and protein sequence alignments were done with DNA STAR (Madison, WI, USA). Sequence homology searches were performed using the NCBI database server. Protein homology was based on BlastX searches [97]. Phylogenetic analysis was performed using the web-based tool at www.phylogeny.fr [98]. The nucleotide and protein sequences were deposited in GenBank under accession number HE614113 (*ppt1*), HE614114 (*aar1*), HE614115 (*fet1*), HE614116 (*ftr1*), HE614117 (*ftr2*), HE614118 (*fet2*), HE614119 (*ftr3*), HE614120 (*fet3*), HE614121 (*nps2*), HE614122 (*nps6*) and HE614123 (*sre1*), respectively.

### Plasmid construction

The putative *F. fujikuroi ppt1* gene and flanking regions were amplified using the primer pairs ppt1-F and ppt1-R which were based on the putative *F. verticillioides ppt1* (FVEG_01894.3) sequence. The *F. fujikuroi ppt1* knock-out plasmid pΔppt1 was created by sequentially cloning the 550 bp 5′ Ff*ppt1* flank (generated with primers ppt1-5′F/ppt1-5′R) and the 770 bp 3′ Ff*ppt1* flank (generated with primers ppt1-3′F/ppt1-3′R) into pNR1 using SacI/XbaI and HindIII/SalI restriction sites, respectively [99], such that the nourseothricin resistance cassette was flanked by *F. fujikuroi* genomic sequence. For generating a complementation construct, a 1.8 kb fragment including 5′ and 3′ non-coding regions was amplified with primers ppt1-Prom-F/ppt1-Term-R. The plasmids pΔaar1, pΔftr3/fet3 and pΔsre1 were assembled using yeast recombinational cloning as essentially described for *Neurospora crassa* deletion vectors [100] and recently established for *F. fujikuroi* vectors [84]. The 5′ and 3′ flanks of Ff*ftr3/fet3* and Ff*sre1* were amplified using primer pairs “gene”-5′-F1/-R1 and “gene”-3′-F1/-R1, respectively. Plasmid DNA from *S. cerevisiae* was extracted using the GeneJET™ Plasmid Miniprep Kit (Fermentas GmbH, St. Leon-Rot, Germany) with slight modifications: cells were resuspended in 300 µl Resuspension Solution plus 100 µl glass beads, lysed by addition of 600 µl Lysis Solution and neutralized with 450 µl Neutralization Solution. DNA fragments used for deletion of Ff*ftr3/fet3* and Ff*sre1* were prepared by PCR using primers “gene”-5′-F1 and “gene”-3′-R1 and 1 µl of pΔftr3/fet3 or pΔsre1, respectively, as template. The plasmid pHphDsRed was constructed by ligating the HindIII/XbaI fragment of pChap-GFP [101] containing the hygromycin resistance cassette into HindIII/XbaI restricted pPgpd-DsRed [102].

### Fungal transformations

Preparation of protoplasts from *F. fujikuroi* mycelium was carried out as described [103]. Briefly, 10^7^ protoplasts of *F. fujikuroi* strains were transformed with 10 µg of the replacement cassette of the vector pΔppt1 or PCR products obtained of pΔftr3/fet3 and pΔsre1, respectively, as described above. Transformed protoplasts were regenerated for 6–7 days at 28°C in a complete regeneration agar (0.7 m sucrose, 0.05% yeast extract) containing 100 µg/ml nourseothricin and 1 mm lysine in case of targeted Ff*ppt1* deletion (Werner-Bioagents, Jena, Germany) or 100 µg/ml hygromycin and 1 mm lysine (Calbiochem, Darmstadt, Germany) in case of targeted Ff*aar1*, Ff*ftr3*/*fet3* and *sre1* deletion. For complementation of ΔFf*ppt1* strains, 10 µg of the genomic Ff*ppt1* PCR fragment was used for transformation as described above, but without addition of lysine in the regeneration media. Protoplasts of the IMI58289 wild-type strain and the Ff*ppt1* mutant were transformed with 20 µg pHphDsRed and transformed protoplasts were regenerated in complete regeneration agar containing 100 µg/ml hygromycin and 1 mm lysine.

The homologous integration events in transformants targeting replacement of Ff*ppt1* with the nourseothricin resistance marker were verified by PCR using primers ppt1- F and ppt1- R targeting outside the replacement fragment in combination with pLOF-OliP and Tub-T, respectively. In case of hygromycine resistant transformants, targeted replacement was verified using a primer outside the replacement fragment in combination with a primer targeting the hygromycine resistance cassette (“gene”-F1d/pCSN44-trpC-T and “gene”-R1d/pCSN44-trpC-P) in case of ΔFf*aar1*, ΔFf*ftr3*/*fet3* and ΔFf*sre1*. The absence or presence of the wild-type gene loci in deletion and add-back strains was verified by PCR using primer pairs targeting the replaced coding region (“gene”-WT-F1/-R1).

### Virulence assays

Infection assays of single plants of *Oryza sativa* spp. *japonica* c.v. Nipponbare were performed as described previously [84]. Gamborg B5 Medium (Duchefa Biochemie, Haarlem, The Netherlands) solution was supplemented with 10 mm lysine and 10 mm BPS when indicated. Microscopy was performed using an Axio Imager.M2 (Carl Zeiss MicroImaging GmbH, Jena, Germany). DIC was used for bright field images and DsRed fluorescence was detected using filterset 38 (excitation band pass 470/40 nm, color splitter 495, emission band pass 525/50 nm). Images were captured with uniform exposure time using an AxioCam MRm and were processed uniformly using AxioVision Rel. 4.8 (both Carl Zeiss MicroImaging GmbH, Jena, Germany).

### Chemical analysis

Gibberellic acids GA_3_ and GA_4/7_ were extracted from 20 ml culture filtrate after 7 days of incubation in ICI medium containing 6 mm glutamine. Extraction was performed using Sep Pak C18 cartridges (Waters GmbH, Eschborn, Germany) from which GA_3_ was eluted with 2 ml 20% acetonitril (ACN) (LGC/Promochem GmbH, Wesel, Germany) and GA_4/7_ were eluted with 2 ml 55% ACN. GA_3_ and GA_4/7_ amounts were measured by HPLC-DAD analysis using a Merck-Hitachi System (Merck KGaA, Darmstadt, Germany) consisting of a gradient pump (L-7100), an autosampler (L-7200) and a Diode Array Detector (L-245). As column a Lichrospher 100 RP-18 column (5 µm; 250 mm×4 mm; Merck KGaA) was applied. HPLC conditions were as follows: solvent A: 0.05% H_3_PO_4_ (Merck KGaA), pH 3; solvent B: ACN. The subsequent gradient was applied: 15 min 15% B; in 20 min to 40% B; in 2 min to 15% B. Data analysis was carried out using EZChrom Elite Version 3.3.2 SP1 (Scientific Software, Inc.). Quantification of GAs was performed by generating a calibration line using different dilutions of 1 mg/ml GA_3_ and GA_4/7_ standards (DKSH GmbH Hamburg, Germany). GA amounts were calculated per 1 l culture filtrate and 1 g dry weight applying peak areas of the different samples and the gradient of the calibration line.

Fusarin, bikaverin and fusarubin production was analyzed as previously described [9], [104].

For moniliformin analyses fungal cultures were extracted as previously described [105] for 1 h on a rotary shaker at 190 rpm at 28°C. For analyses 1 ml of extract was evaporated under a stream of nitrogen at 40°C, dissolved in 150 µl 5% Methanol (v/v) and analyzed by HPLC-FTMS using chromatographic conditions as previously described [106].

The volatiles released by agar plate cultures were collected and analyzed as previously described [8]. Briefly, the volatiles emitted by the fungal cultures on solidified CM were collected by use of a closed loop stripping apparatus (CLSA). Therefore, a circulating air flow was directed through a charcoal filter (Chromtech GmbH, Idstein, Precision Charcoal Filter, 5 mg) in a closed apparatus containing the fungal culture for 24 h. The charcoal filter was extracted with 30 µl of analytically pure dichloromethane and the obtained solutions were immediately analyzed by GC-MS and stored at −80°C. GC-MS analyses were carried out on a HP6890 GC system connected to a HP5973 Mass Selective Detector fitted with a HP-5 fused silica capillary column (25 m×0.22 mm, 0.25 µm film, SGE Inc.). Conditions were as follows: inlet pressure: 77.1 kPa, He 23.3 ml min^−1^; injection volume: 1 µl; injector: 250°C; transfer line: 300°C; electron energy: 70 eV. The GC was programmed as follows: 50°C (5 min isothermic), increasing at 10°C min^−1^ to 320°C, and operated in splitless mode (60 s valve time); carrier gas (He): 1.0 ml min^−1^. Quantification was carried out by peak integration with the MSD Chem Station software (Agilent) of three replicate samples and is given as arithmetic means ± standard deviations, normalized to 100% for the production of the C-1995 wild-type strain.

## Supporting Information

Figure S1 **Deletion strategy of Ff** ***ppt1*** **and Southern blot analysis.** A: Gene replacement of Ff*ppt1*. Physical maps of the SacI/ApaI gene replacement fragment from the plasmid pΔppt1, the *Ffppt1* locus from the wild-type strain IMI58289 and the gene locus from a *Ffppt1* knock-out mutant showing the nourseothricin resistance cassette (grey). Small arrows indicate positions of primers used for cloning the replacement vector and for the PCR analysis of replacement mutants. Dotted lines and Roman numerals represent primer combinations used for the diagnostic PCR shown in B. B: Diagnostic PCR results of the analyzed Ff*ppt1* replacement transformants and the wild type (WT) as well as the complemented strains ΔFf*ppt1* ^C^. Roman numerals represent primer combinations as schematically drawn in A. M: marker in kb. C: For the Southern blot analysis the genomic DNA of the wild type and Δ*Ffppt1* strains was digested with EcoRI, blotted and hybridized with the HindIII/SalI flank of the replacement vector pΔppt1 as probe (heavy line with asterisks). In three mutants the wild-type fragment with a size of 18.5 kb is replaced by a 4.8 kb fragment, resulting from an additional EcoRI restriction site in the nourseothricin resistance cassette. M: marker in kb.
(TIF)

Figure S2 **Diagnostic PCR results of gene replacement transformants.** A: Diagnostic PCR results of the analyzed Ff*aar1* replacement transformants and the wild type (WT). Roman numerals represent primer combinations as schematically drawn. M: marker in kb. B: Diagnostic PCR results of the analyzed Ff*ftr3/fet3* replacement transformants in wild-type and ΔFf*ppt1* background, respectively. Roman numerals represent primer combinations as schematically drawn. M: marker in kb. C: Diagnostic PCR results of the analyzed Ff*sre1* replacement transformants and the wild type (WT). Roman numerals represent primer combinations as schematically drawn. M: marker in kb.
(TIF)

Figure S3 **Influence of FfPpt1, FfSre1 and FfFtr3/FfFet3 on growth on extreme iron conditions.** Growth of indicated mutants on solidified complete medium (CM) without iron (10 mm Lys) and 1 mm FeCl_3_. Representative pictures were taken after 3 days of incubation at 28°C in darkness.
(TIF)

Figure S4 **Influence of FfPpt1 on growth on lysine-deficient media.** Representative photographs of indicated strains on solidified CD media supplemented with or without lysine as indicated.
(TIF)

Figure S5 **Influence of FfPpt1 on sexual mating recognition.** A, C: Representative photographs of sexual crossings of indicated strains as described in Methods. Scale bar represents 1 cm. B: Representative magnifications of sexual crossings seen in A showing produced perethicia. Scale bar represents 1 cm. D: Representative photographs of sexual crossings of indicated strains as described in Methods on media supplemented with FEC. Scale bar represents 1 cm.
(TIF)

Figure S6 **Influence of FfPpt1 on secondary metabolite gene expression and moniliformin production.** A: Northern blot analysis of all six bikaverin cluster genes in the designated strains and rRNA visualization as loading control. B: Northern blot analysis of Ff*pks6* in the designated strains and rRNA as loading control. C: Extracted ion chromatogram of moniliformin detected by HPLC-FTMS as described in Methods. Black: MRC2276; blue: Ff*ppt1* mutant in MRC2276.
(TIF)

Table S1 **Primer used in this study.**
(DOCX)
