# Supplementary figures and images for: The Sfp-Type 4′-Phosphopantetheinyl Transferase Ppt1 of Fusarium fujikuroi Controls Development, Secondary Metabolism and Pathogenicity
Source: PLoS One. 2012 May 25;7(5):e37519. doi: 10.1371/journal.pone.0037519 (PMC3360786; doi:10.1371/journal.pone.0037519)

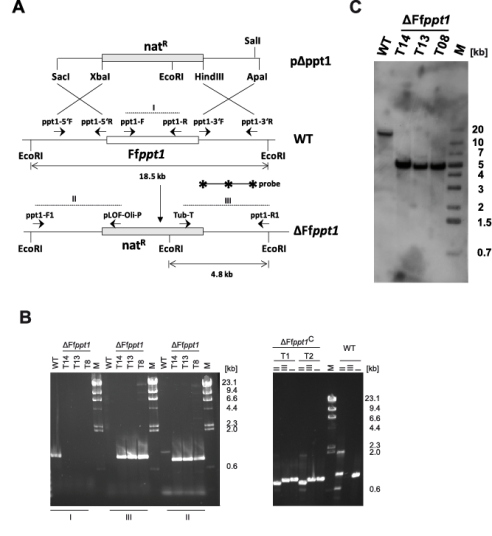

Supplement: Figure S1 — Deletion strategy of Ff ppt1 and Southern blot analysis. A: Gene replacement of Ffppt1. Physical maps of the SacI/ApaI gene replacement fragment from the plasmid pΔppt1, the Ffppt1 locus from the wild-type strain IMI58289 and the gene locus from a Ffppt1 knock-out mutant showing the nourseothricin resistance cassette (grey). Small arrows indicate positions of primers used for cloning the replacement vector and for the PCR analysis of replacement mutants. Dotted lines and Roman numerals represent primer combinations used for the diagnostic PCR shown in B. B: Diagnostic PCR results of the analyzed Ffppt1 replacement transformants and the wild type (WT) as well as the complemented strains ΔFfppt1 C. Roman numerals represent primer combinations as schematically drawn in A. M: marker in kb. C: For the Southern blot analysis the genomic DNA of the wild type and ΔFfppt1 strains was digested with EcoRI, blotted and hybridized with the HindIII/SalI flank of the replacement vector pΔppt1 as probe (heavy line with asterisks). In three mutants the wild-type fragment with a size of 18.5 kb is replaced by a 4.8 kb fragment, resulting from an additional EcoRI restriction site in the nourseothricin resistance cassette. M: marker in kb. (TIF) [file pone.0037519.s001.tif]

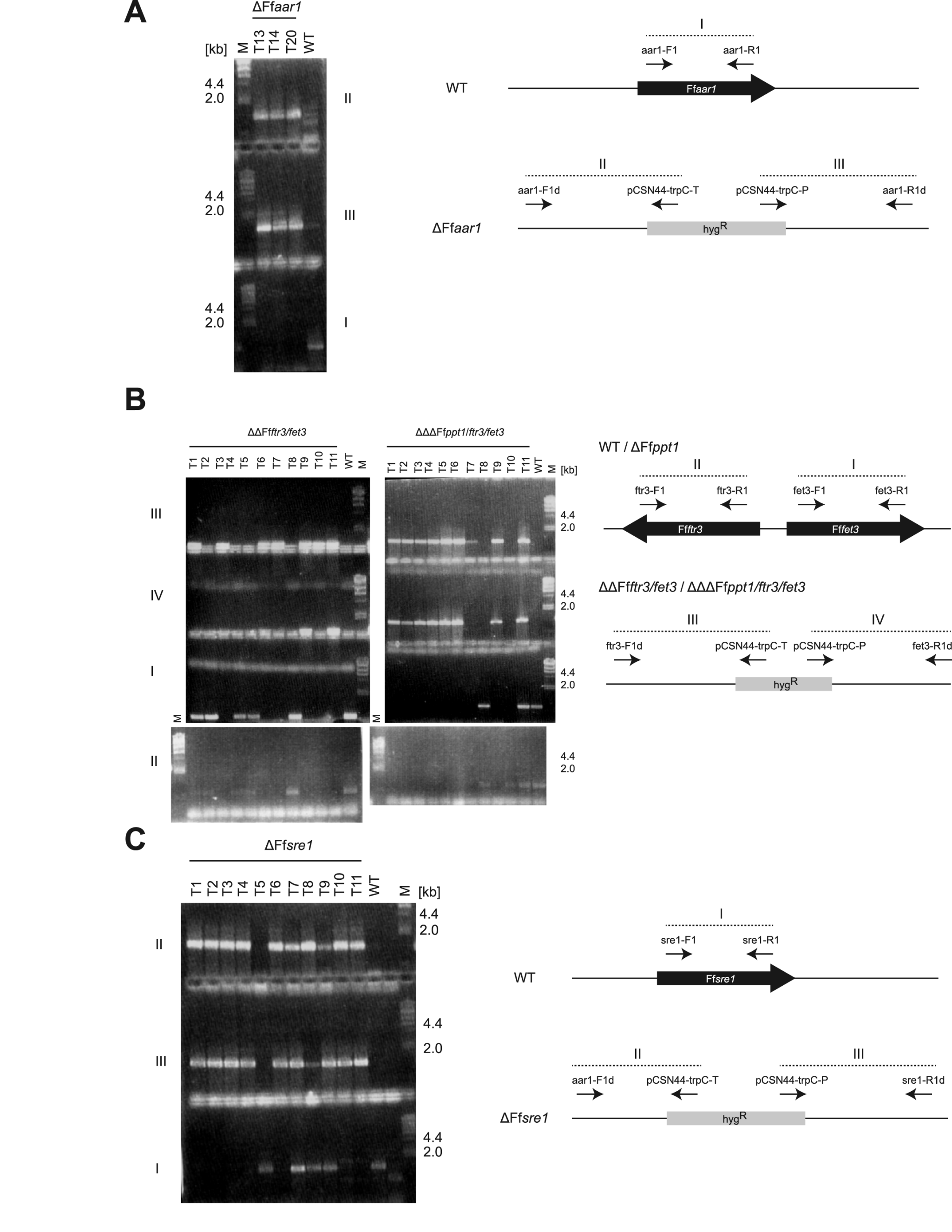

Supplement: Figure S2 — Diagnostic PCR results of gene replacement transformants. A: Diagnostic PCR results of the analyzed Ffaar1 replacement transformants and the wild type (WT). Roman numerals represent primer combinations as schematically drawn. M: marker in kb. B: Diagnostic PCR results of the analyzed Ffftr3/fet3 replacement transformants in wild-type and ΔFfppt1 background, respectively. Roman numerals represent primer combinations as schematically drawn. M: marker in kb. C: Diagnostic PCR results of the analyzed Ffsre1 replacement transformants and the wild type (WT). Roman numerals represent primer combinations as schematically drawn. M: marker in kb. (TIF) [file pone.0037519.s002.tif]

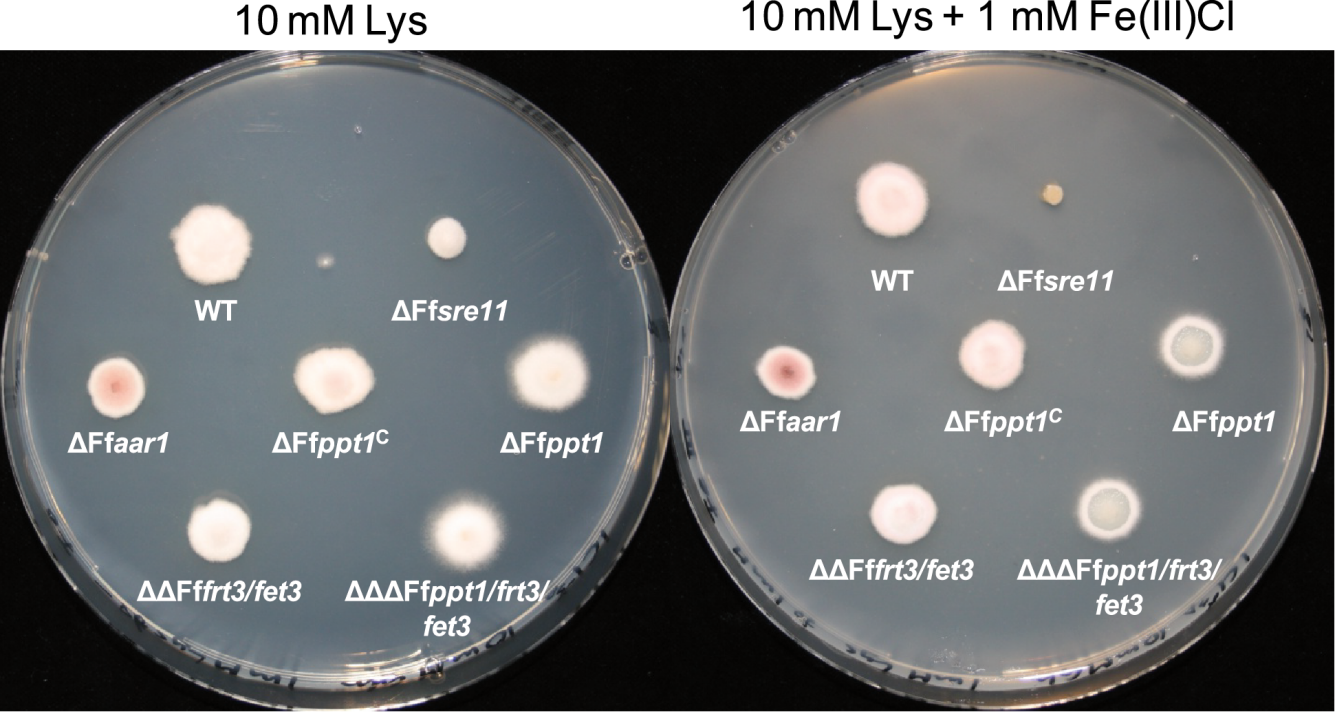

Supplement: Figure S3 — Influence of FfPpt1, FfSre1 and FfFtr3/FfFet3 on growth on extreme iron conditions. Growth of indicated mutants on solidified complete medium (CM) without iron (10 mm Lys) and 1 mm FeCl3. Representative pictures were taken after 3 days of incubation at 28°C in darkness. (TIF) [file pone.0037519.s003.tif]

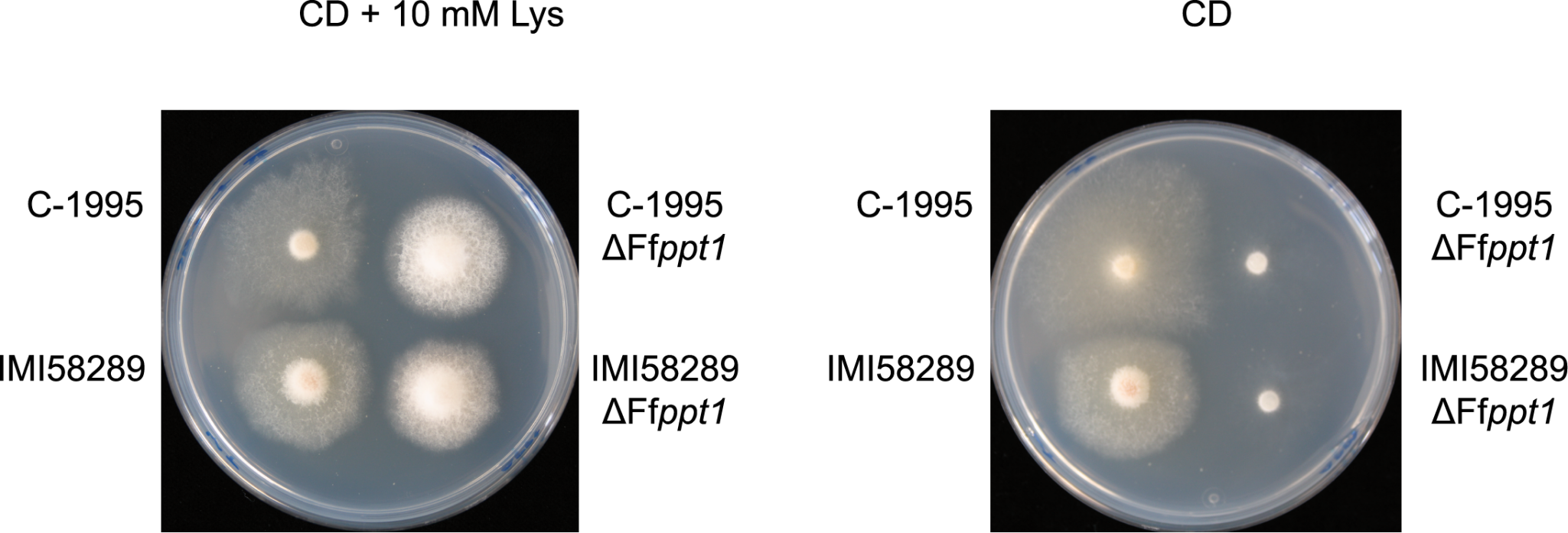

Supplement: Figure S4 — Influence of FfPpt1 on growth on lysine-deficient media. Representative photographs of indicated strains on solidified CD media supplemented with or without lysine as indicated. (TIF) [file pone.0037519.s004.tif]

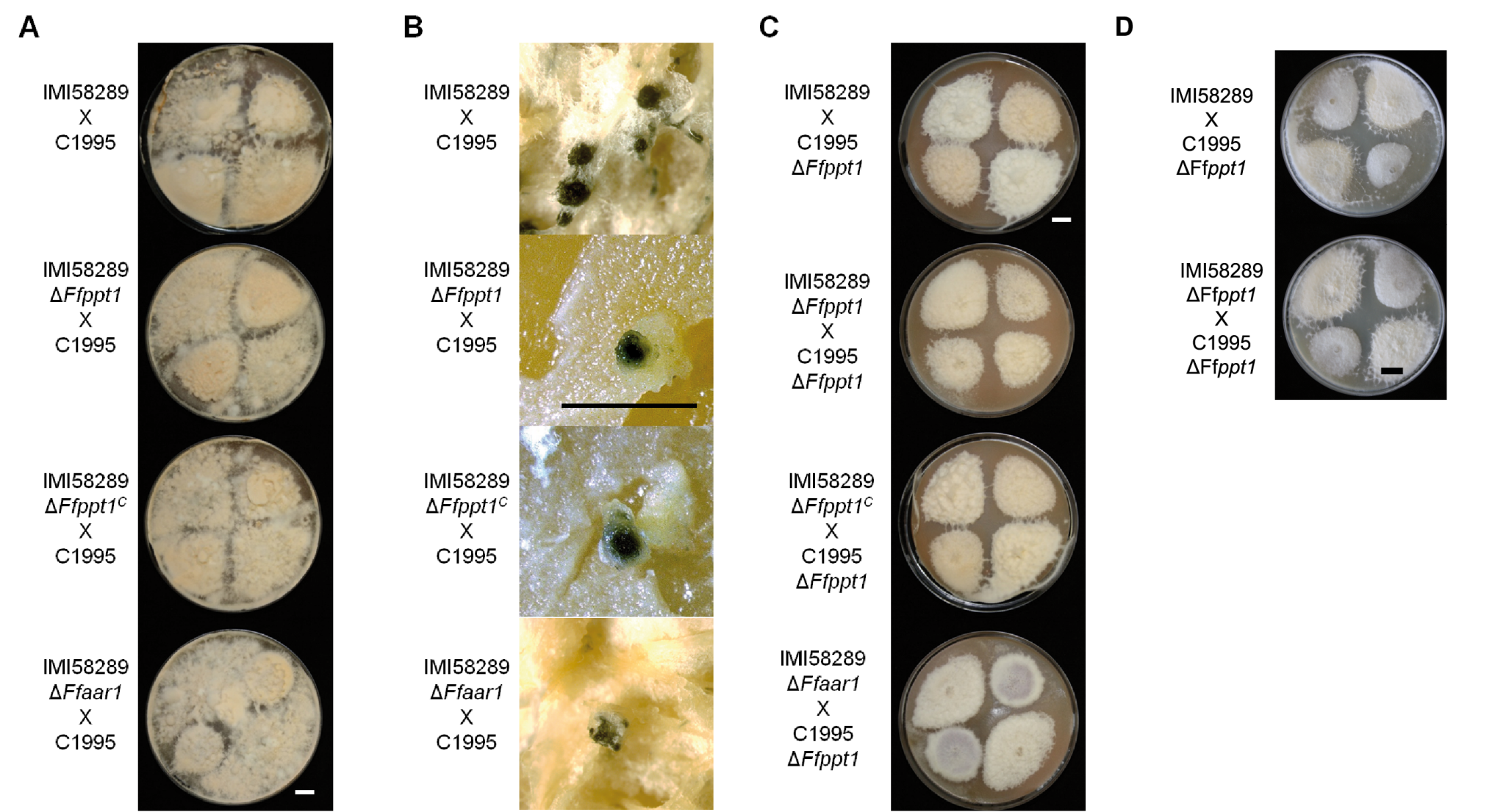

Supplement: Figure S5 — Influence of FfPpt1 on sexual mating recognition. A, C: Representative photographs of sexual crossings of indicated strains as described in Methods. Scale bar represents 1 cm. B: Representative magnifications of sexual crossings seen in A showing produced perethicia. Scale bar represents 1 cm. D: Representative photographs of sexual crossings of indicated strains as described in Methods on media supplemented with FEC. Scale bar represents 1 cm. (TIF) [file pone.0037519.s005.tif]

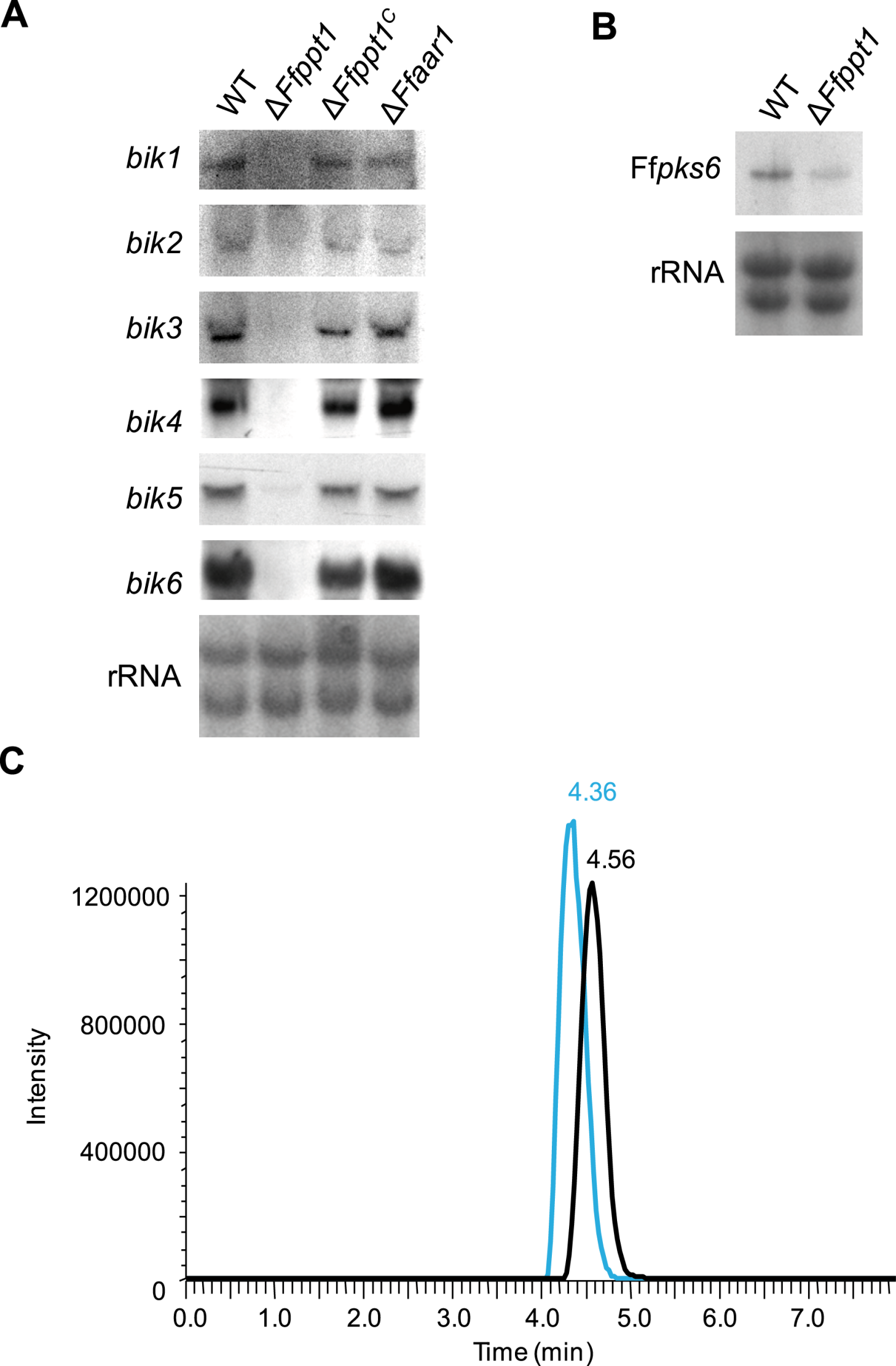

Supplement: Figure S6 — Influence of FfPpt1 on secondary metabolite gene expression and moniliformin production. A: Northern blot analysis of all six bikaverin cluster genes in the designated strains and rRNA visualization as loading control. B: Northern blot analysis of Ffpks6 in the designated strains and rRNA as loading control. C: Extracted ion chromatogram of moniliformin detected by HPLC-FTMS as described in Methods. Black: MRC2276; blue: Ffppt1 mutant in MRC2276. (TIF) [file pone.0037519.s006.tif]
