# Supplementary material for: The Sfp-Type 4′-Phosphopantetheinyl Transferase Ppt1 of Fusarium fujikuroi Controls Development, Secondary Metabolism and Pathogenicity
Source: PLoS One. 2012 May 25;7(5):e37519. doi: 10.1371/journal.pone.0037519 (PMC3360786; doi:10.1371/journal.pone.0037519)
Supplement: Table S1 — Primer used in this study. (DOCX) [file pone.0037519.s007.docx]

**Supporting Table 1. Primer used in this study.**

| ppt1-5‘F | GAAAGGAACTGAGCVTCCGGTAGATACACTGG |
| --- | --- |
| ppt1-5‘R | CTATTGCCTTGGAATGCTCTAGAGCTAGC |
| ppt1-3‘F | CATGATCAGAGAAGCTTTTATGAATAATGAAAC |
| ppt1-3‘R | CTAGTCGACGATGTAAGTGCTCCCATCATCATCTGTTCG |
| ppt1-F | GGAGTTTTGTTGTGCCCACAC |
| ppt1-R | GATCAGACTCTTCAGGGAGCTGACATTC |
| ppt1-Prom-F | GCATGCTAAGAGTGCACCTTATGTTTCCGG |
| ppt1-Term-R | ATAGTATTGCTGGGGTGGCAAGGTCGTTGC |
| ppt1-F1 | GTTGCAGTAACGATGAGTAGGG |
| ppt1-R1 | GTTCACCAAATGCCAATATTTC |
| pLOF-Oli-P | GGTACTGCCCCACTTAGTGGCAGCTCGCG |
| Tub-T | GGTCCTCGGAGTGGAGAGGG |
| pCSN44-trpC-T | GGAATAGAGTAGATGCCGACCGG |
| pCSN44-trpC-P | CCTCCACTAGCTCCAGCCAAGCCC |
| aar1-F1d | CTTGAGACATGCGGCTTGTGAG |
| aar1-R1d | CAATCGCGACAGGTGTTGCGG |
| sre1-F1d | GTCTCGGATACCCTTCTCCC |
| sre1-R1d | GATGCTCAACTACTCCACGC |
| ftr3-F1d | GGTGATTGTGTTGGAGGAAGG |
| fet3-R1d | GTCAAGCCACCAAGCCAGCC |
| sre1-5F | GTAACGCCAGGGTTTTCCCAGTCACGACGCTTGGCTCTGCTTGGTCACC |
| sre1-5R | ATCCACTTAACGTTACTGAAATCTCCAACGTCTGGGTTGGAATGCGATGG |
| sre1-WT-F1 | CGGCGCGAACAGCGCTGCC |
| sre1-WT-R1 | GGCGACAGAAACGAGGCAGG |
| sre1-3F | CTCCTTCAATATCATCTTCTGTCTCCGACGCAAGGTATCACTGGACGCG |
| sre1-3R | GCGGATAACAATTTCACACAGGAAACAGCGGATATATCATGAATGGCCC |
| ftr3/fet3-5R | GCGGATAACAATTTCACACAGGAAACAGCGGTTCGGTGATGTGCTAAAACC |
| ftr3/fet3-5F | CTCCTTCAATATCATCTTCTGTCTCCGACGCGATATGGTAACCTTGGCG |
| ftr3/fet3-3F | ATCCACTTAACGTTACTGAAATCTCCAACGGTGTTTAGCTGCGTGGCGGG |
| ftr3/fet3-3R | GTAACGCCAGGGTTTTCCCAGTCACGACGCGGCATGGGCTATGAGACGG |
| ftr3-WT-F1 | CCCAGACGGCTTTGCTGAAGAGACC |
| ftr3-WT-R1 | GTCTCGAAACAAGCATCATCGTCTCCG |
| fet3-WT-F1 | GGATCTGACGATGAACAACCTCGG |
| fet3-WT-R1 | CCGGGAGAGGAGGCACCATC |
| aar1-5F | GTAACGCCAGGGTTTTCCCAGTCACGACGGGTGGGCTTAAAAGAGTAGAG |
| aar1-5R | ATCCACTTAACGTTACTGAAATCTCCAACCGTCTCCATTCTGGATGCCGG |
| aar1-3F | CTCCTTCAATATCATCTTCTGTCTCCGACGATGATAGGAACGCTGTGGCTG |
| aar1-3R | GCGGATAACAATTTCACACAGGAAACAGCGTAGGCTGCGGGTGAAGGACG |
| aar1-WT-F1 | GCGTGTCCTGGGCCGTATG |
| aar1-WT-R1 | CGATGGTGACATCAGCACGC |
| hph-F | GTCGGAGACAGAAGATGATATTGAAGGAGC |
| hph-R | GTTGGAGATTTCAGTAACGTTAAGTGGAT |
